# Recombinant* Lactococcus lactis* Expressing Haemagglutinin from a Polish Avian H5N1 Isolate and Its Immunological Effect in Preliminary Animal Trials

**DOI:** 10.1155/2017/6747482

**Published:** 2017-02-22

**Authors:** Agnieszka K. Szczepankowska, Katarzyna Szatraj, Przemysław Sałański, Agnieszka Rózga, Roman K. Górecki, Jacek K. Bardowski

**Affiliations:** Institute of Biochemistry and Biophysics, Polish Academy of Sciences, Warsaw, Poland

## Abstract

Lactic acid bacteria (LAB) are Gram-positive, nonpathogenic microorganisms that are gaining much interest as antigen producers for development of live vaccine vectors. Heterologous proteins of different origin have been successfully expressed in various LAB species, including* Lactococcus lactis*. Recombinant* L. lactis* strains have been shown to induce specific local and systemic immune responses against various antigens. Our study aimed at constructing a* L. lactis* strain expressing haemagglutinin of a Polish avian H5H1 influenza isolate and examining its effect on animals. Expression of the cloned* H5* gene was achieved using the nisin-controlled gene expression system. Detection of the intracellular H5 antigen produced in* L. lactis* was performed by Western blot analysis and confirmed using mass spectrometry. The potential of* L. lactis* recombinant cells to induce an immune response was examined by setting up preliminary immunization trials on chickens and mice. Obtained sera were tested for specific antibodies by ELISA assays. The results of these studies are a promising step toward developing a vaccine against the bird flu using* Lactococcus lactis* cells as bioreactors for efficient antigen production and delivery to the mucosal surface.

## 1. Introduction

*Lactococcus lactis* are noninvasive, nonpathogenic bacteria, used widely in manufacturing of milk fermentation products. Lack of lipopolysaccharide (LPS) and enterotoxins and the generally acknowledged food-grade status make* L. lactis* cells a useful tool for bioengineering processes. In the last several decades lactococci have been exploited as hosts for expression of heterologous antigen proteins, including those of therapeutic and prophylactic activity [1, 2]. Among them, a* L. lactis*-based interleukin-10 (IL-10) formulation has been subjected to phase II clinical trials of inflammatory bowel disease (IBD) therapy [3]. Latest studies focus on employing these bacteria as live vaccine delivery systems to mucosal surfaces by oral or intranasal routes [4–6]. Mucosal epithelial cells constitute an entry for infectious agents, including influenza viruses. It is expected that vaccines directed to mucosal surfaces will induce immune responses, providing both mucosal and systemic protection. Moreover, advantages of mucosally administered versus traditional (parenteral) vaccinations include low cost and easy and painless antigen dosage, which is especially beneficial for large-scale immunizations of farm animals or human populations. Mucosally administered vaccines based on recombinant* L. lactis* cells producing various antigens were shown to evoke specific immune responses of humoral and cell-mediated type [7–10]. At the same time, it has been demonstrated that certain* L. lactis* strains (e.g., NZ9000) exhibit innate adjuvant properties, which can enhance specific immune response to administered antigens [11–13]. This feature is advantageous especially that mucosally applied vaccine preparations alone usually present low immunogenicity [14, 15]. Additionally, the general inability of* L. lactis* as noncommensal, noncolonizing bacteria to grow or replicate in vivo limits its potential to evoke oral tolerance [8, 16]. Furthermore,* L. lactis*-produced antigens are presented to the immune system in particulate form, which is postulated to evoke stronger mucosal response than soluble proteins [17, 18]. The great potential of* L. lactis* cells has been exploited by ActoGeniX™, which is one of the first companies to develop recombinant biologically contained strains (termed ActoBiotics™) for clinical purposes [3].

In our study, we cloned the codon-optimized gene encoding haemagglutinin of the H5N1 influenza virus and expressed it intracellularly using the nisin-controlled gene expression (NICE®) system in* L. lactis* NZ9000 strain. The strain carries two signal transduction genes,* nisK* and* nisR,* integrated into the chromosome, which regulate the nisin-induced promoter PnisA present in the vector. The system allows efficient but controlled expression of recombinant genes in* L. lactis* cells, including toxic proteins, on lab and industrial scales [19, 20].

Our study was based on the* HA* gene of the H5N1 A/swan/Poland/305-135V08/2006 virus isolated from a wild swan during a highly pathogenic avian influenza outbreak in 2006 in Poland. Due to the realistic threat of an epidemic spread (for the first time in this country), development of an efficient and safe vaccine for protection of local farm poultry flocks based on the H5 antigen from this specific isolate was greatly sought. Haemagglutinin (HA) is the most abundant and immunogenic protein on the surface of the influenza virus which plays role in the initial steps of host infection [21]. It was shown to induce specific antibodies and is by far the most widely used antigen in designing human and animal vaccines against flu [22–24]. Also in chickens, the HA protein of the avian influenza virus (AIV) was shown to elicit specific immune response [25]. Although AIV develops in birds as natural hosts, interspecies infections, including human, have also been reported. Based on the statements of the World Human Organization (WHO), more than 50% of confirmed human cases of H5N1 led to death in years 2003–2016 [26]. Transmission of AIV to humans has been linked to domestic birds, such as chickens [23]. Therefore, the risk of interspecies barrier break and effective AIV infection in humans and its further spread in the human population is apparent. For that reason, developing a strategy of fast and cheap production of protective anti-H5N1 vaccine for wide-scale immunization of farm birds is strongly desirable.

Our results, based on preliminary animal trials, show that it is possible to elicit an immune response by oral delivery of live H5-producing* L. lactis* cells. Specific antibody titers were examined in ELISA assays to assess the efficacy of the vaccination strategy. Variations in response between examined animal species (chickens and mice) were determined. The work provides data that sets a basis for developing an effective vaccine formulation for birds using recombinant* L. lactis* against H5N1 infection.

## 2. Materials and Methods

### 2.1. Bacterial Strain, Vector, and Growth Conditions

*Lactococcus lactis* NZ9000 strain and pNZ8150 (MoBiTec) were used, respectively, as the antigen expression host and plasmid vector. Bacterial cells were cultured at 30°C in M17 (Oxoid) broth [27] containing 0.5% glucose (GM17) without shaking or on GM17 plates with 1.5% agar. For selection of pNZ8150-carrying strain and its recombinant variants, chloramphenicol (Cm) was added to the growth medium at a concentration of 10 *μ*g ml^−1^. Prior to their use, bacterial cells for immunization trials were suspended in PBS, pH 7.4, supplemented with 20% (v/v) trehalose to maintain viability during storage and postthaw handling.

### 2.2. Sequence Modification and Cloning of* H5* Gene in* L. lactis*

Gene encoding the H5 nucleocapsid protein of the avian influenza virus H5N1 A/swan/Poland/305-135V08/2006 strain from AIV EpiFlu Database [http://platform.gisaid.org] (accession number: EP1156789) was commercially synthesized by GeneArt™. For stability, DNA fragment corresponding to the protein's proteolytic cleavage site (ΔRRRKKR aa) was deleted from the original nucleotide sequence. For efficient translation and mRNA stability in* L. lactis* cells, codon optimization was performed by modifying the* H5* nucleotide sequence using the GeneOptimizer® software. The codon-optimized* H5* nucleotide sequence was amplified by PCR reaction using FHASnisScaI/RHASSacI (5′ ATGGAAAAAATTGTTCTT 3′/5′ GCC**GAGCTC**GTTAAATACAAATACG 3′) primers and Pfu DNA Polymerase (Thermo Fisher Scientific) generating blunt ends. The 5′ end of the reverse primer was adapted to contain the SacI restriction site (in bold). Next, the generated PCR fragment was subjected to SacI digestion (Thermo Fisher Scientific) and then blunt-sticky end ligation with pNZ8150 cut previously with SacI/ScaI enzymes. In effect, a translational fusion with the* nisA* promoter was obtained. The resulting recombinant plasmid was termed pNZ8150:H5 and introduced into* L. lactis* NZ9000 cells via electroporation.

### 2.3. DNA Manipulations, Transformation of Bacterial Cells, and Identification of Recombinant Clones

DNA molecular cloning and restriction enzyme analysis were performed according to general procedures [28].* L. lactis* NZ9000 strain cells were made electrocompetent for transformation as already described [29, 30]. Transformants were selected on GM17 plates supplemented with Cm and further analyzed by “colony PCR” using primers nisF/nisR (5′AACTACAAAATAAATTATAAGGAGGCACTC3′/5′GTTTCAAGCCTTGGTTTTCTAATTTTGG3′). Plasmid DNA was isolated using PureYield™ Plasmid Miniprep system (Promega). Prior to the isolation step, the cell suspension was incubated in TES buffer (25 mM Tris-HCl, pH 8, 10 mM EDTA, and 1% saccharose) with lysozyme (8 mg ml^−1^) for 30 min at 37°C to disrupt the peptidoglycan layer and increase the efficiency of plasmid DNA recuperation. Obtained recombinant construct was subjected to DNA restriction analysis. Finally, nucleotide sequence of the cloned DNA fragment was confirmed by sequencing (GS FLX Titanium 454, Roche). The resulting* L. lactis* NZ9000 strain carrying the pNZ8150:H5 recombinant vector was termed NZ-H5.

### 2.4. Immunodetection

Intracellular production of H5 in recombinant bacteria was examined by growing NZ-H5 cells at 30°C on GM17 (Oxoid) medium supplemented with Cm (10 *µ*g ml^−1^). At log-phase (OD_600_ 0.4), nisin (5 ng ml^−1^) was added to induce gene expression. Culture samples were harvested 1, 2, and 3 h after induction. Subsequently, crude extracts were obtained by disrupting the cells 5x for 45 sec with 1.5 min intervals on ice using the Mini-BeadBeater (BioSpec Products) and glass beads (106 *μ*m diameter; Sigma). To inhibit potential degradation, each sample was combined with a protease inhibitor according to the manufacturer's instruction (Roche). Cellular debris and glass beads were removed by centrifugation for 10 min at 8,000 rpm and 4°C. Protein extracts were combined with an equal volume of 2x loading buffer (125 mM Tris-HCl, pH 6.8, 4% SDS, 10%  *β*-mercaptoethanol, 20% glycerol, and 0.01% bromophenol blue), heated for 5 min at 95°C. Next, protein samples were separated on a 12% polyacrylamide gel by SDS-PAGE (120 V, 500 mA) and subsequently transferred onto nitrocellulose Hybond-C Extra membrane (Amersham). The membrane was blocked overnight at 4°C with TBS-T buffer (20 mM Tris, pH 8, 150 mM NaCl, and 0.1% Tween 20), containing 3% skimmed milk. Immunoblotting was carried out using commercial monoclonal anti-H5 mouse antibodies (Abcam) at 1 : 500 dilution, followed by detection with secondary anti-IgG mouse antibodies conjugated with alkaline phosphatase (Sigma) at dilution 1 : 15,000. Immunoblots were resolved using NBT/BCIP Stock Solution (Roche) in ALT (1 ml 1 M Tris, pH 8.0, 1 ml 1 M NaCl, and 0.5 ml 1 M MgCl_2_) buffer. 293 cell culture-purified Qinghai-HA(H5N1)(A/Bar-headed Goose/Qinghai/12/05) protein (Immune Technology) was used as a positive control. Data was normalized using protein samples from cells carrying the empty vector (NZ [pNZ8150]).

### 2.5. Mass Spectrometry and Protein Identification

Proteomic analyses presented in this work were performed in the Laboratory of Mass Spectrometry at IBB PAS. Briefly, gel-excised protein samples were reduced with 100 mM DTT (30 minutes at 56°C) and alkylated with 0.5 M iodoacetamide (45 minutes in a darkroom at RT), followed by trypsin (10 ng *µ*l^−1^, Promega) overnight digestion at 37°C. Peptide mixtures were concentrated, desalted on a RP-C18 precolumn (Waters), and separated on a nano-Ultra Performance Liquid Chromatography (UPLC) RP-C18 column (Waters, BEH130 C18 column, 75 *µ*m i.d., 250 mm long), using a 160 min gradient from 5 to 30% of acetonitrile. Measurements were performed with the Orbitrap Velos spectrophotometer (Thermo Fisher Scientific), working in the regime of data-dependent MS to MS/MS switch with HCD type peptide fragmentation. Identification of proteins was performed using the Mascot search engine (Matrix Science) with the probability-based algorithm. Data were searched with automatic decoy database and filtered to obtain a false discovery rate below 1%.

### 2.6. Animal Immunization and Sera Collection

Leghorn White laying type chickens were housed in cages in an experimental poultry house under standard commercial conditions. Female BALB/c mice (supplied by Mossakowski Medical Research Centre of the Polish Academy of Sciences, Warsaw, Poland) were kept in a temperature-controlled environment at 24°C with 12 h day-night cycles and received food and water ad libitum.

Vaccination was performed using a prime-boost strategy. One-day-old chickens or eight-week-old mice were immunized orally using a gauge syringe with 10^9^ CFU of NZ-H5 cells or respective amount of cell lysates (L-H5). Cells carrying the empty vector (NZ[pNZ8150]) and crude cell extract (L-pNZ8150) served as respective negative controls. Doses were administered on days 1–3 and then again as two boosters at two-week intervals (on days 7–9 and 21–23 for chickens or days 14–16 and 28–30 for mice). Blood samples from the wing veins (chickens) or orbital sinus (mice) were collected on day 20 and day 38 (day of termination) for chickens and day 9, 17, 25, and 41 (day of termination) for mice. To obtain serum, whole blood samples were left to coagulate at room temperature for approximately 15 to 30 minutes. Then, samples were centrifuged twice at 4°C for approximately 10 min at 10,000*g* and stored at −80°C. Mice feces samples (0.1 g) for IgA detection were collected on days 0, 9, 17, 25, and 41 and combined with 1 ml of PBS buffer containing protease inhibitor at a given concentration (Roche). Samples were vigorously vortexed and then centrifuged at 10,000*g* for 10 min at 4°C. The supernatant was collected and kept at −80°C until further use.

### 2.7. Enzyme-Linked Immunosorbent Assay

Sera from immunized animals were subjected to ELISA assays for detection of specific IgY (chickens) and IgG and IgA (mice). In short, MaxiSorp plates (Nunc) were coated with recombinant H5 protein of A/Bar-headed Goose/Qinghai/12/05 (H5N1) strain of influenza A virus (3 *μ*g ml^−1^ in PBS, overnight, 2–8°C) (Immune Technology). The next day, serum samples were diluted 1 : 25 in 2% BSA-PBS, applied onto plates, and incubated overnight at 2–8°C. Proper controls and blanks (sample diluent) were included. Secondary labeling and detection were done with Sigma-Aldrich reagents unless specified otherwise. Antibody classes were detected with goat (HRP)-labeled antibodies against chickens IgY at 1 : 2500 dilution or against mouse IgG and IgA at 1 : 500 dilution (Thermo Fisher Scientific) (1 h, 37°C). Tetramethylbenzidine (TMB) was used as a substrate. Reactions were performed at room temperature for 30 min and then stopped by adding 0.5 M sulfuric acid. Absorbance was read at 450 nm with subtraction of the mean OD readings of blanks. Samples were determined to be positive for anti-H5 antibodies when OD readings were 2 SD above the mean OD of samples from sham-immunized animals (cut-off value).

## 3. Results

### 3.1. Recombinant H5 Expression in* L. lactis* NZ9000

**Figure 1:**
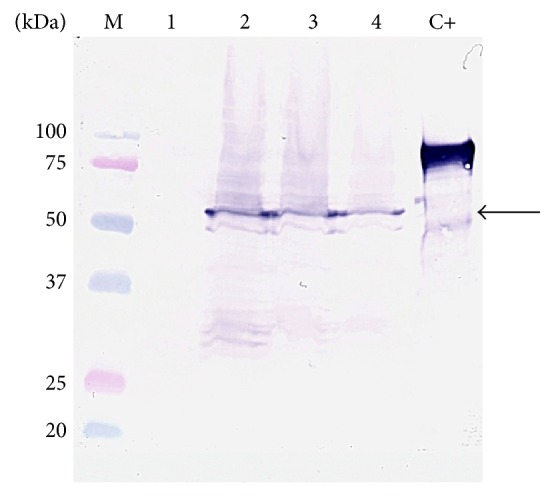
Immunoblot detection of H5 production under* nisA* promoter in* L. lactis* NZ9000 cells at different induction times. M: marker, Precision Plus Protein Dual Color Standards (Bio-Rad). Protein extracts: lane 1, NZ[pNZ8150] (negative control); lanes 2, 3, and 4, NZ-H5 after nisin (5 ng ml^−1^) induction for 1, 2, or 3 hrs, respectively. Qinghai-HA(H5N1)(A/Bar-headed Goose/Qinghai/12/05) protein (Immune Technology) was used as a positive control (C+). The migration level of bands corresponding to detected H5 recombinant protein (63 kDa) is marked by a black arrow.

The codon-optimized* H5* gene was cloned under the* nisA* promoter in pNZ8150 vector and introduced to* L. lactis* NZ9000 generating the recombinant NZ-H5 strain. Log-phase cultures of NZ-H5 and the negative control (NZ[pNZ8150]) were induced for intracellular antigen production by addition of nisin and further growth for 1, 2, or 3 hrs. After disruption of collected cultures, crude cell extracts were subjected to SDS-PAGE followed by immunoenzyme reaction using specific H5 antibodies. Antigen-specific signals corresponding to the full-length protein (63 kDa) were identified in all NZ-H5 samples collected at different times after induction (Figure 1, lanes 2, 3, and 4) and were absent for the NZ[pNZ8150] control (Figure 1, lane 1). Obtained results confirmed intracellular production of H5 in* L. lactis* cells under the tested nisin concentration and times of induction.

### 3.2. H5 Identification by Mass Spectrometry

**Figure 2:**
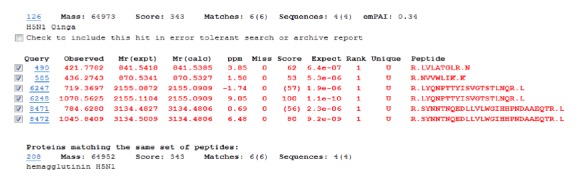
Peptides detected by mass spectrometry from H5 immunoreactive gel-excised band.

To confirm that the observed signal corresponds to the H5 protein, the 63 kDa immunoreactive band was excised from the SDS-PAGE gel and subjected to mass spectrometry analysis. Detected peptides were identified as fragments of the H5 protein. Obtained results were consistent with Western blot analysis, confirming antigen production in* L. lactis* cells (Figure 2).

### 3.3. Study of H5-Specific Immune Responses in Chickens and Mice

**Figure 3:**
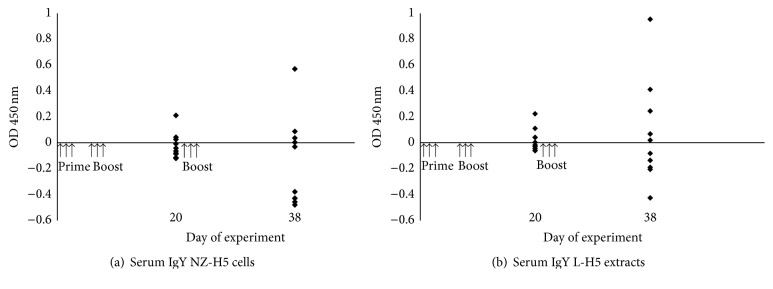
Specific serum antibody response in chickens fed with* L. lactis* NZ-H5 or respective cell lysates L-H5. Animals (*n* = 10) were given 10^9^ CFU of bacteria (a) or respective amounts of cell lysates (b) on days marked by arrows. H5-specific IgY titers were measured in serum samples collected on days 20 and 38 at 1 : 25 dilution. Graphs show results obtained for each immunized chicken at given time point of blood sampling (diamonds). Data was normalized by adapting the cut-off (point 0 on the axis) as mean OD readings higher by 2 SD than for control birds.

To assess the immunoefficiency of recombinant H5 produced in* L. lactis*, preliminary vaccination trials were performed on chickens. Birds were fed with NZ-H5 cells, L-H5 lysates, or respective controls (NZ[pNZ8150] or L-pNZ8150) using an established prime-boost scheme. Antigen-specific IgY titers were measured approximately 3 and 5 weeks after prime immunization (Figures 3(a) and 3(b)). NZ-H5 cells elicited immunoresponse in three out of ten chickens tested. In one bird, IgY titer was higher than in others and further increased after the second boosting (Figure 3(a)). Compared to the NZ-H5 group, chickens vaccinated with L-H5 showed a significantly higher level of specific IgY antibodies (Figure 3(b)). In this case, half of the birds responded three weeks after the initial immunization. This effect was maintained after the second booster with a further increase in IgY titer.

To examine whether more eminent immune responses can be obtained with recombinant* H5*-expressing* L. lactis* cells, a well-adapted laboratory mouse model was used, and necessary negative controls were applied (NZ[pNZ8150] or L-pNZ8150). For animals vaccinated with NZ-H5 cells, anti-HA IgG and IgA titers were just above or below the threshold level (Suppl. Figure 1 in Supplementary Material available online at https://doi.org/10.1155/2017/6747482). Alternatively, higher levels of specific antibodies were noted after administration of L-H5 lysates (Suppl. Figure 2). Here, several mice responded positively giving a rise in IgG isotype. For one animal, the most profound IgG peak was noted 9 days after the first booster (day 25) (Suppl. Figure 2A). Two mice responded by a raise of both IgG and IgA. Highest IgA titers were observed 11 days after the second booster (day 41) (Suppl. Figure 2B). Nonetheless, these results conclude that the applied immunization scheme could elicit only a very weak immunoresponse in mice under the tested conditions.

## 4. Discussion

The use of* L. lactis* cells as live delivery vectors has gained much interest over the last years as an alternative method of vaccination. Such approach is especially attractive with regard to prevention of massive disease spreads in animals, including avian influenza outbreaks. It excludes the necessity of traditional vaccination based on manual injection, which in case of numerous bird flocks can be quite laborious. Administration of antigens produced in* L. lactis* to gut mucosa via the oral route is reported to be effective in triggering both local and systemic immune responses [4, 31]. It is postulated that mucosally administrated vaccines via oral or nasal routes demand adjuvants to elicit specific protective responses. In this aspect of vaccine development,* L. lactis* cells seem to be an attractive antigen producer as they were shown to have intrinsic adjuvant properties [12]. Specifically for the lactococcal NZ9000 strain, recent in vitro studies demonstrate its potential to evoke immune response by induction of cytokines and maturation of dendritic cells in bone marrow [32].

In this work, we evaluated the efficiency of expression of haemagglutinin of the avian influenza H5N1 A/swan/Poland/305-135V08/2006 isolate in* L. lactis* cells. In our previous work, we have shown that it is possible to engineer* L. lactis* cells to produce the H5 protein of the avian influenza virus H5N1 [33]. In this study, we aimed at further improvement of the approach. To reduce the potential protein toxicity, expression of* H5* was carried out under the nisin-inducible promoter (PnisA) using the NICE system [19]. Its tight regulation allows for stable expression of proteins of various function and origin [34–38]. Western blot assays revealed the presence of an immunospecific band of 63 kDa, irrelevant of induction time, which corresponded to H5. The size of the recombinant H5 (63 kDa) was slightly different from that of the H5 Qinghai reference protein, which migrated at the level of the 75 kDa band of the protein marker. This size difference is most probably related to the protein producer (*L. lactis* versus eukaryotic cell line, resp.). Posttranslational modifications (e.g., glycosylation and phosphorylation), known to occur in eukaryotic cells, which are often lacking in prokaryotic hosts (in* L. lactis* cells glycosylation was not determined), may contribute to the molecular weight variations of these two H5 proteins. Therefore, mass spectrometry analysis was conducted and has confirmed the identity of the protein. The obtained data clearly evidence the production of the H5 protein of the avian influenza virus H5N1 in* L. lactis* cells. Immunogenicity of the recombinant antigen was preliminarily screened using two animal models. In mice, very low IgG and IgA antibody titers were detected in single animals. More enhanced levels of specific anti-H5 antibodies (IgY) were detected in chickens. The most pronounced effect was noted for birds ingested with preparations containing crude extracts L-H5. Variations in response between animal species, at least in part, reflect the acknowledged diversity of immunological systems of birds and mice (mammals) [39]. Moreover, the receptor binding profiles of avian versus mammalian (human) type A influenza viruses are different [40, 41]. While haemagglutinin of human viruses participates in recognition of receptors in the respiratory tract (*α*2-6-galactose linked sialic acids), avian viruses bind to receptors in intestinal epithelium cells (*α*2-3-galactose linked sialic acids) [42]. It is feasible that the divergence in discerning* L. lactis*-produced H5 by epithelial cells of the two species accounts for variations in the response observed during animal immunization trials.

The objective of the study was to design a biological* L. lactis*-based platform for H5 protein production and its easy delivery to gut mucosa. Intracellular localization of the protein was intended in order to protect the antigen during passage through the stomach before reaching the intestine. This approach was envisaged as advantageous especially for large-scale immunizations of farm animals, such as chickens, where the recombinant* L. lactis* could be administered in food or drinking water. As fate of recombinant* Lactococcus* cells (degree and rate of lysis) was hard to predict, animals were also immunized with whole-cell lysate preparations (L-H5). ELISA assays revealed differences in the levels of specific antibody titers depending on the type of immunization preparation. The higher number of chickens responding in detectable IgY titers after administration of whole-cell extracts (L-H5) suggests that in this form the antigen is more immunogenic. It is probable that even higher overall specific antibody titers could be obtained by designing recombinant cells, where the H5 antigen is anchored to the cell wall. A series of studies confirmed that cell surface-exposed antigens in* L. lactis* are produced at higher levels, are more stable, and elicit stronger immune responses after mucosal vaccination than their cytoplasmic forms [43–46]. In* L. lactis* cells, internally localized heterologous proteins are often degraded due to activity of proteolytic proteins, which potentially can account for a weaker answer from the immune system [47, 48]. Another crucial aspect in development of an efficient immune response is protein folding and exposition of specific conformational epitopes. The inability to identify the recombinant H5 under nondenaturing Western blot conditions (data not shown), suggests altered protein conformation (although other unknown cellular factors influencing this state cannot be excluded) with respect to the control protein. This may impact the antibody titers detected in ELISA assays. From another point of view, low serum antibody titers may also suggest poor exposition of the cell-enclosed protein to gut mucosa. Despite the anticipated breakage of* L. lactis* cells, it cannot be excluded that the generally lower effect of recombinant NZ-H5 is additionally due to the presence of trehalose in the bacterial preparations. Trehalose is a cryoprotectant, known to stabilize biological membrane bilayers [49]. The intention to use trehalose was to protect the cells from freeze/thaw handling. The compound was also anticipated to protect recombinant bacteria from gastric acid before reaching the gut as noted by Bahey-El-Din [50]. Our results suggest that trehalose may reduce cell lysis also in further parts of the GI tract, which is reflected by higher anti-HA specific antibody titers for lysate (L-H5) versus recombinant cell preparations.

In the course of this study, we determined that the H5 antigen from the avian influenza virus H5N1 can be produced in* L. lactis* cells. Preliminary oral animal immunizations revealed a difference in the response levels between different species. Our study clearly shows that it is of significant importance to test potential vaccine formulations on target hosts as direct extrapolation of immunization effects performed on model animal species may not necessarily be valid. Moreover, we achieved higher antibody responses with crude cell extracts (L-H5) rather than intracellular localized protein (NZ-H5). Under the tested immunization schemes, fluctuations in specific serum titers were observed between individual chickens. However, in positively responding birds, anti-HA antibody levels increased after subsequent boosting, indicating that a properly developed immune reaction can be maintained during the time course of the study. Further modifications of the immunization scheme are anticipated to determine the most effective procedure of dosing the recombinant bacterial preparations. Overall, production of H5 in* L. lactis* cells and the observed immune response after oral administration of recombinant bacteria are a starting point in developing an effective preparation for immunizing birds against avian flu.

## Supplementary Material

Supplementary Figure 1. Specific serum antibody response in mice ingested with *L. lactis* NZ-H5 cells. Days on which mice (*n* = 10) were immunized with 109 CFU of bacteria are indicated by arrows. H5-specific IgG (A) and IgA (B) titers were measured in serum samples collected on days 9, 17, 25 and 41 at 1:25 dilution. Graphs show results obtained for each immunized mice at given time point of blood sampling (diamonds). Data was normalized by adapting the cut-off (point 0 on the axis) as mean OD readings higher by 2 SD than for control mice. Supplementary Figure 2. Specific serum antibody response in mice ingested with *L. lactis* L-H5 cell lysates. Days on which mice (*n* = 10) were immunized with 109 CFU of bacteria are indicated by arrows. H5-specific IgG (A) and IgA (B) titers were measured in serum samples collected on days 9, 17, 25 and 41 at 1:25 dilution. Graphs show results obtained for each immunized mice at given time point of blood sampling (diamonds). Data was normalized by adapting the cut-off (point 0 on the axis) as mean OD readings higher by 2 SD than for control mice.
